# Sildenafil Versus Placebo for Early Pulmonary Vascular Disease in Scleroderma (SEPVADIS): protocol for a randomized controlled trial

**DOI:** 10.1186/s12890-024-02892-3

**Published:** 2024-04-30

**Authors:** Matthew R. Lammi, Monica Mukherjee, Lesley Ann Saketkoo, Kyle Carey, Laura Hummers, Steven Hsu, Amita Krishnan, Marie Sandi, Ami A. Shah, Stefan L. Zimmerman, Paul M. Hassoun, Steven C. Mathai

**Affiliations:** 1 Louisiana State University Health Sciences, 1901 Perdido St, 70112 New Orleans, LA, USA; 2 Comprehensive Pulmonary Hypertension Center, University Medical Center, New Orleans, USA; 3 New Orleans Scleroderma and Sarcoidosis Patient Care and Research Center, New Orleans, USA; 4 Division of Cardiology, Johns Hopkins University, Baltimore, USA; 5 Tulane University School of Medicine, New Orleans, USA; 6 Institute for Clinical and Translational Medicine, Johns Hopkins University, Baltimore, USA; 7 Johns Hopkins University, Division of Rheumatology, Baltimore, USA; 8 Johns Hopkins University, Department of Radiology and Radiological Science, Baltimore, USA; 9 Johns Hopkins University, Division of Pulmonary and Critical Care Medicine, Baltimore, USA

**Keywords:** Pulmonary hypertension, Scleroderma, Systemic sclerosis, Clinical trial, Sildenafil

## Abstract

**Background:**

Pulmonary hypertension (PH) is a leading cause of death in patients with systemic sclerosis (SSc). An important component of SSc patient management is early detection and treatment of PH. Recently the threshold for the diagnosis of PH has been lowered to a mean pulmonary artery pressure (mPAP) threshold of > 20 mmHg on right heart catheterization (RHC). However, it is unknown if PH-specific therapy is beneficial in SSc patients with mildly elevated pressure (SSc-MEP, mPAP 21–24 mmHg).

**Methods:**

The SEPVADIS trial is a randomized, double-blind, placebo-controlled phase 2 trial of sildenafil in SSc-MEP patients with a target enrollment of 30 patients from two academic sites in the United States. The primary outcome is change in six-minute walk distance after 16 weeks of treatment. Secondary endpoints include change in pulmonary arterial compliance by RHC and right ventricular function by cardiac magnetic resonance imaging at 16 weeks. Echocardiography, serum N-terminal probrain natriuretic peptide, and health-related quality of life is being measured at 16 and 52 weeks.

**Discussion:**

The SEPVADIS trial will be the first randomized study of sildenafil in SSc-MEP patients. The results of this trial will be used to inform a phase 3 study to investigate the efficacy of treating patients with mild elevations in mPAP.

**Trial registration:**

ClinicalTrials.gov Identifier NCT04797286.

## Background

Pulmonary hypertension (PH) is a hemodynamic condition defined by directly measured pulmonary arterial pressures from right heart catheterization (RHC). Until recently, an individual was considered to have PH when their mean pulmonary artery pressure (mPAP) was ≥ 25 mmHg at rest. In 2018, the 6th World Symposium on Pulmonary Hypertension (WSPH) lowered the threshold for a diagnosis of PH to a mPAP > 20 mmHg on resting RHC [1]. This recommendation was based on prior data describing the normal mPAP as 14 ± 6 mmHg [2], with 20mmHg therefore representing the 95th percentile of mPAP. Additionally, multiple large epidemiological studies demonstrated that there is a continuum of risk whereby a mPAP ≥ 19 mmHg is strongly associated with worse survival [3, 4]. A meta-analysis of 8 studies comprising almost 12,000 patients found that there is a 34–78% increased risk of death in those with a mPAP of 19–24 mmHg compared to those with a normal mPAP [5].

The updated definition of PH is most relevant to populations at high-risk for pulmonary hypertension who undergo routine screening. One such group of patients are those with systemic sclerosis (SSc, also known as scleroderma), who suffer from a severe autoimmune disease characterized by exaggerated fibrosis, vasculopathy, and dysregulation of the immune system that can affect the skin and internal organs. The prevalence of mild increases in mPAP (21–24 mmHg) in SSc, hereafter referred to as SSc with mildly elevated pulmonary pressures (SSc-MEP), is 10–15% [6–9]. This prevalence is similar to the prevalence of pulmonary arterial hypertension (PAH) in SSc, a form of group 1 PH with significant morbidity and mortality, when defined using the mPAP ≥ 25 mmHg cut-off [10]. Application of evidence-based PH early detection strategies such as the DETECT algorithm led to an increase in the identification of SSc patients with a mildly increased mPAP [11]. Therefore, improved early detection strategies and the changed definition of PH may significantly increase the number of SSc patients who now have PH. Compared to SSc patients with a mPAP < 21 mmHg, SSc-MEP patients have worse exercise capacity with a lower six-minute walk distance (6MWD) and peak exercise workload, along with impaired right ventricular (RV) output reserve during exercise [8], demonstrating that SSc-MEP patients have significant functional limitations.

While epidemiologic data show increased morbidity and mortality for persons with mildly elevated pulmonary artery pressures and guidelines now support lower thresholds for establishing the diagnosis of PH, there are no approved therapies for treatment of patients with this form of PH, which may substantially impact survival and progression to more severe PH. Only one prior trial has attempted to address this patient population. The EDITA study was a randomized controlled trial (RCT) of an endothelin receptor antagonist (ERA), ambrisentan versus placebo in 38 SSc subjects who either had SSc-MEP or an exercise-induced increase in mPAP [12]. Although the primary endpoint, change in mPAP after 6 months of treatment, was not different between the groups, the ambrisentan-treated participants had an improvement in resting pulmonary vascular resistance (PVR) and cardiac index (CI) change during exercise. Change in 6MWD also favored the ambrisentan group, with a 39-meter (m) improved compared to placebo [12]. This trial demonstrated the potential for a PAH medication to improve outcomes in SSc patients with mildly elevated mPAP but was limited by the mixed nature of the cohort and the selection of their primary endpoint, since mPAP is unlikely to change significantly due to the narrow range of pressures in SSc-MEP.

The lack of data examining the utility of vasodilator therapy in the management of SSc-MEP motivated the “**S**ildenafil Versus Placebo for **E**arly **P**ulmonary **V**ascular **D**isease **I**n **S**cleroderma” (SEPVADIS) Study. This study is a RCT of sildenafil versus placebo in SSc-MEP patients to investigate the following aims: (1) To determine whether sildenafil affects the 6MWD in SSc-MEP patients at 16 weeks and 1 year; (2) To determine if sildenafil affects RV function in SSc-MEP patients at 16 weeks; (3) To determine whether sildenafil affects health-related quality of life (HRQoL) in SSc-MEP patients at 16 weeks and 1 year.

## Methods

### Design and setting

SEPVADIS is a bicentric randomized, placebo-controlled, double-masked, parallel group superiority trial that is being conducted at two academic PH and SSc referral centers in the United States: Johns Hopkins University (Baltimore, MD) and Louisiana State University Health Sciences Center (New Orleans, LA).

### Participant selection

**Fig. 1:**
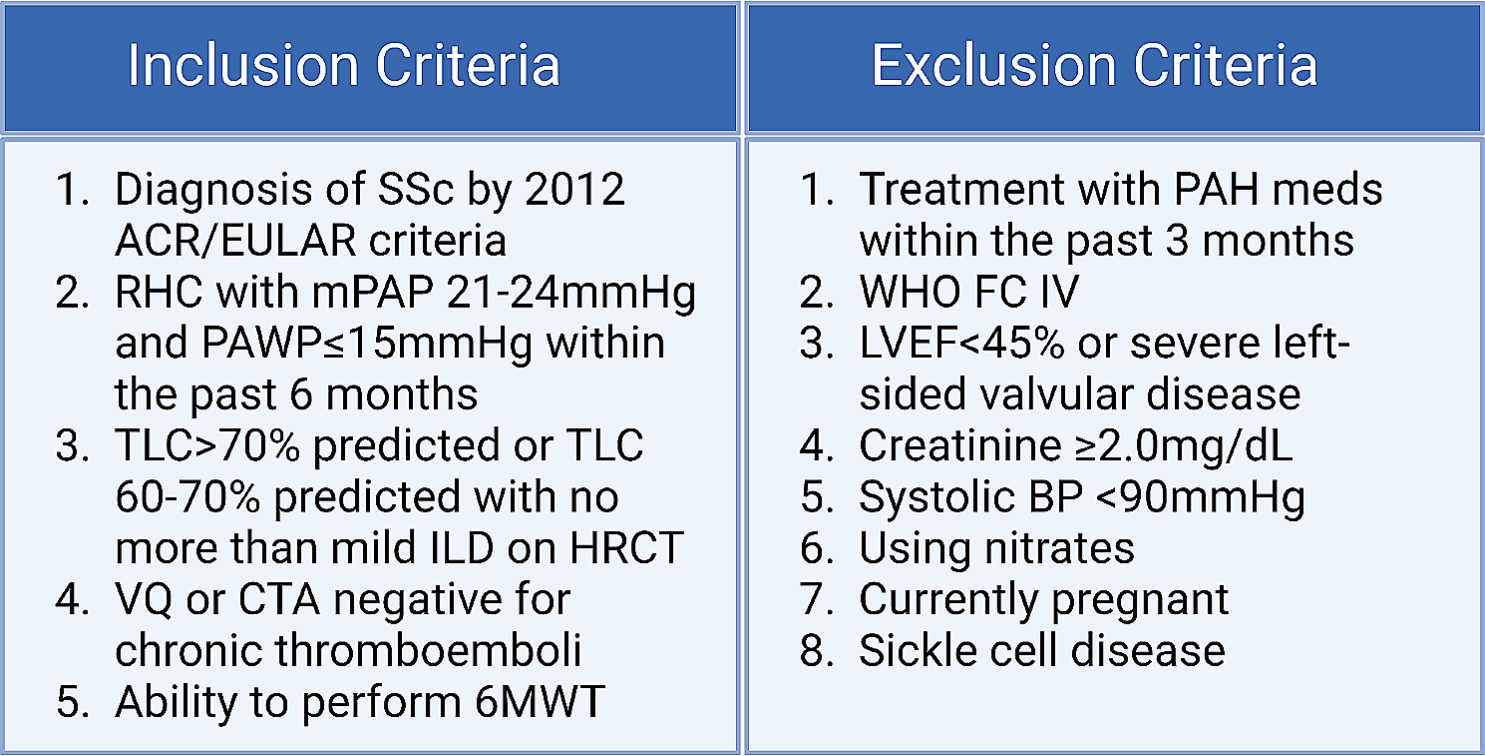
Selection criteria. SSc = systemic sclerosis; ACR = American College of Rheumatology; EULAR = European League Against Rheumatism; RHC = right heart catheterization; mPAP = mean pulmonary artery pressure; PAWP = pulmonary artery wedge pressure; TLC = total lung capacity; ILD = interstitial lung disease; HRCT = high resolution computed tomography; VQ = ventilation-perfusion scan; CTA = chest CT angiography; 6MWT = six-minute walk test; PAH = pulmonary arterial hypertension; WHO = World Health Organization; FC = functional class; LVEF = left ventricular ejection fraction; BP = blood pressure

Recruitment is occurring at each study site, drawing from patient referrals and each center’s existing SSc population. The selection criteria are displayed in Fig. 1. The main inclusion criteria are a diagnosis of SSc and a diagnosis of pre-capillary PH with a mPAP 21–24 mmHg and a pulmonary artery wedge pressure (PAWP) ≤ 15mmHg. Of note, we did not include a PVR > 3 Wood units [1], which has been supported by the latest European guidelines, which lowered the PVR threshold for PAH to > 2 Wood units [13, 14]. Exclusion criteria are listed in Fig. 1 and focus on the exclusion of participants in whom sildenafil would be contraindicated, such as severe systemic hypotension, use of nitrates [15], and sickle cell disease [16].

### Interventions

Sildenafil is a phosphodiesterase-5 (PDE-5) inhibitor that was originally FDA-approved for the treatment of erectile dysfunction, but was later investigated for PAH in the SUPER trial, in which sildenafil led to improvements in 6WMD, mPAP, and functional class [17]. In a sub-group of connective tissue disease PAH, most of whom had SSc, significant benefit was seen with sildenafil [18]. This medication is used for SSc-related Raynaud’s phenomenon and digital ulcers and has been proven to be safe in a group of SSc patients with a mPAP < 25mmHg [19]. We chose a PDE-5 inhibitor rather than an ERA due to an RCT that demonstrated improved 6MWD, RV mass, and HRQoL in PAH patients who received sildenafil compared to an ERA [20]. Additionally, in patients with SSc-PAH initial treatment with PDE-5 inhibitor monotherapy was associated with less clinical worsening compared to ERA [21].

Sildenafil is being purchased from Teva Pharmaceuticals, who are not involved in the planning or conduct of this trial. Participants are randomized to either sildenafil (20 mg) or matching placebo taken three times per day. Sildenafil and placebo tablets are being over-encapsulated by the Research Pharmacy at the Johns Hopkins University (JHU) School of Medicine Research Pharmacy. At the Research Pharmacy, capsules are being packaged into bottles with a liner, cotton, and childproof cap. One bottle of drug product is dispensed to study subjects at the baseline study visit and at the 16-week visit during the treatment phase. Subjects are asked to return bottles at the 16-week and 52-week visits to allow for tracking of adherence and medication control. Randomization to drug or placebo is done in a 1:1 fashion blocked and stratified by center. All study personnel, subjects, and the statistician are blinded for the duration of the study until the last subject completes follow-up assessments. The JHU Research Pharmacist is unblinded. In the rare event that unmasking is necessary for clinical care, the Chair of the Steering Committee will make the decision to unmask, and the treating physician will call the research pharmacy to obtain the participant’s treatment assignment. Subjects will be withdrawn from the trial if the participant withdraws consent or if the principal investigator determines that the subject should be withdrawn for safety. One specific scenario in which this could occur is if the participant has a mPAP > 25mmHg and a PAWP ≤ 15mmHg on their week 16 RHC; they would be withdrawn from the trial and given guideline-based PAH treatment.

### Outcomes

The primary outcome of this trial is 6MWD measured at 16 weeks. Six-minute walk distance reflects peak oxygen consumption [22] in PAH and is associated with changes in HRQoL [23], with a minimal important difference (MID) of 33 m for PAH and 24 m for SSc-PAH [23]. The 16-week timepoint was chosen since nearly every proprietary drug for PAH has been approved based on changes in 6MWD at 3–4 months as this is considered a clinically important intermediate endpoint [17, 24–27]. Second, improvement in 6MWD in SSc-PAH patients can be seen in as few as 8 weeks in treatment-naïve patients [28]. We are following patients for one year and collecting 6MWD along with other data to assess the durability of the effect of sildenafil in SSc-MEP.

Secondary endpoints include change in pulmonary arterial compliance (PAC) and RV function by cardiac magnetic resonance imaging (CMR) and echocardiography, serum N-terminal probrain natriuretic peptide (NT-proBNP), and HRQoL at 16 weeks. Six-minute walk distance, NT-proBNP, HRQoL, and echocardiography are also obtained at 52 weeks. PAC is a measure of pulmonary arterial stiffness and a contributor to RV afterload which, due to the inverse hyperbolic relationship of PVR and PAC [29], may be modifiable early in the course of PAH. This is of significant relevance in SSc-PAH, since PAC is an independent predictor of survival [30]. Echocardiographic measures of RV function include tricuspid annular planar systolic excursion (TAPSE) [31] and speckle tracking echocardiography (STE), a quantitative measurement of regional and global contractility that is responsive to PAH treatment and correlates strongly with functional capacity and hemodynamics [32]. CMR, the gold standard for RV function assessment, is being employed to quantify RV ejection fraction and RV volumes. HRQoL is assessed using the Medical Outcomes Survey Short Form-36 (SF-36) [33] and emPHasis-10, a disease-specific tool developed specifically for PAH [34]. Testing protocols were standardized between the two enrolling sites; RHC, echocardiography, and CMR done at LSU will be interpreted centrally at Johns Hopkins.

### Data collection and participant timeline

**Fig. 2:**
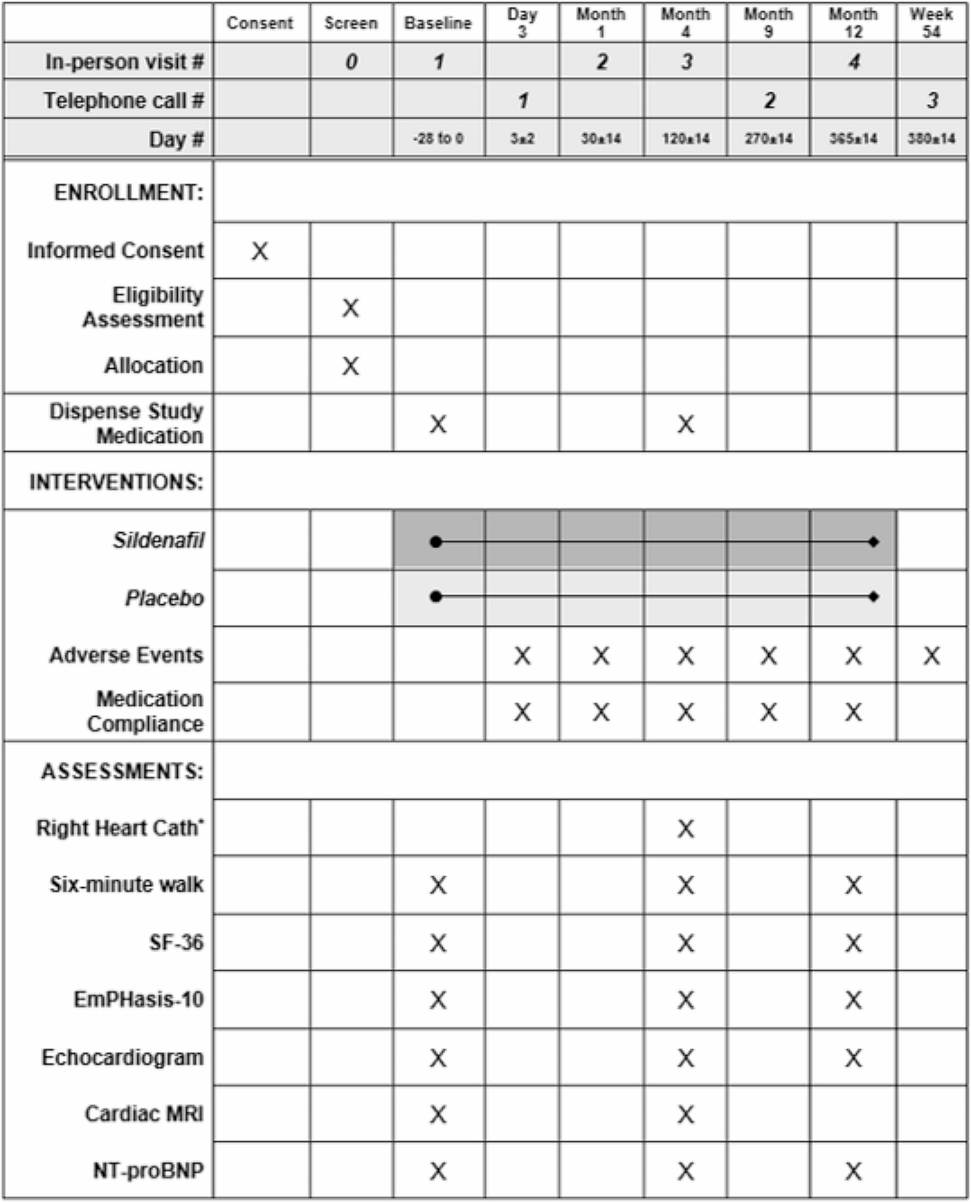
Participant timeline and schedule of events. SF-36 = Short Form 36 questionnaire; MRI = magnetic resonance imaging; NT-proBNP = N-terminal pro B-type natriuretic peptide. ^*^: The right heart catheterization that is used for inclusion into the trial was done prior to enrollment as part of clinical care

The participant timeline is displayed in Fig. 2. Subject retention is being addressed through the following methods: extensive contact information will be recorded for each participant and the research coordinator will call before each study visit to encourage attendance. Additionally, participants are contacted and encouraged by the research coordinator to report any serious adverse events as they occur, while any other adverse event reporting occurs at the next available study visit or scheduled phone check in. Participants are being reimbursed for their time and reasonable travel expenses necessary for their participation. Non-adherence with therapy is being minimized by emphasizing the importance of compliance with study drug treatment and performing pill counts at study visits. If a participant wishes to drop-out from the treatment phase of the study or has a serious adverse event, we will continue to follow-up with the subject for study assessments to assist with safety monitoring and to avoid the problems introduced by missing data. Any missing data that will be reported as a protocol deviation to the single IRB and any other relevant monitoring authorities.

### Specimen collection and storage

In addition to the laboratory tests to be run locally at each visit, research blood is being collected and processed by the research coordinator at each site. Those research collections are processed the same day as the visit and are stored initially at -80 degrees Celsius in freezers at the local site. The processed samples are then transported at a later date to the central JHU Scleroderma Biorepository for long term storage.

### Statistical methods

#### Sample size

**Fig. 3:**
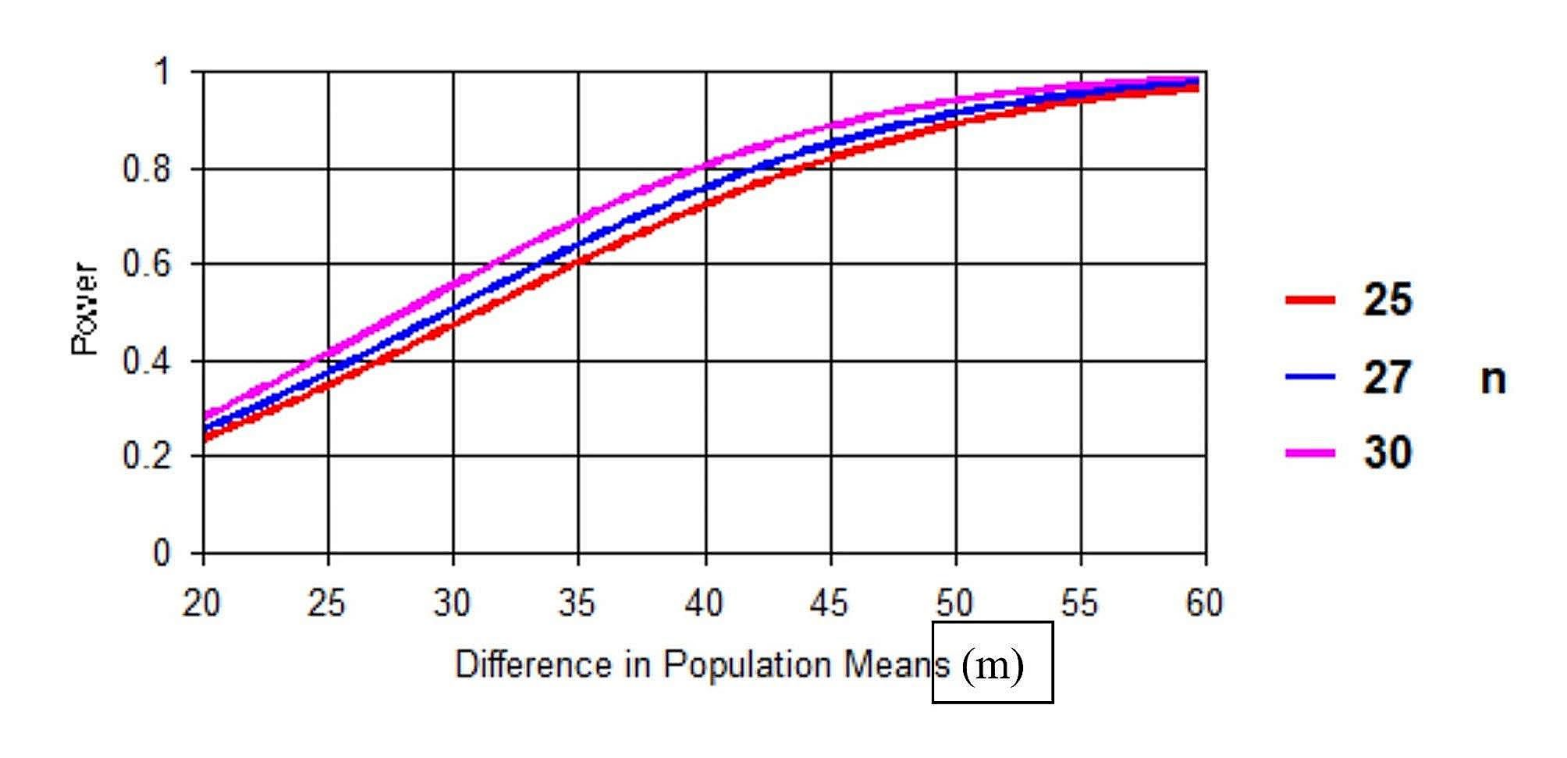
Power estimates and detectable alternatives by sample size in the SEPVADIS trial

We have based our effect size estimate of 45 m upon our preliminary data (not shown) demonstrating a difference in mean 6MWD between SSc patients with normal mPAP and SSc-MEP of ∼ 50 m with a standard deviation of 75 m. As such, we will have > 80% power to detect this difference at significance level of 0.05 with 27 subjects. To account for a 10% drop-out rate, we are enrolling 30 subjects. Even if our actual drop-out rate nearly doubles our expected rate (17% vs. 10%), we maintain adequate power to detect a difference of 45 m with 80% power (Fig. 3). While this estimate of change in 6MWD exceeds the MID for the 6MWD in PAH, the MID for this test in SSc-MEP is unknown. Further, if differences in 6MWD observed in the study do not reach the predefined detectable alternative, clinical relevance of the effect on 6MWD may be reflected by comparing the proportion of patients who exceeded the MID for 6MWD between arms. Based upon a sample size of 27 subjects completing the trial, we also have adequate power (80% or greater) to detect differences in the proposed secondary outcome measures. For instance, we have 80% power to detect a mean difference in PAC of 0.6 mL/mmHg (SD 1.1) between treatment arms; this difference was the average difference between these SSc-MEP and SSc-normal pressures found in our preliminary studies (data not shown). Further, we will have sufficient power to detect differences of 0.23 cm (SD 0.5) in TAPSE between groups which is close to the estimated MID for TAPSE in SSc-PAH (0.22 cm) from prior work from our group [28]. For global RV strain measured by STE, we have more than 85% power to detect a difference of 4.8% (SD 8%); this is the difference detected in our ATPAHSS Study of ambrisentan and tadalafil in treatment naïve SSc-PAH patients after therapy [28, 32]. Similarly, we have adequate power to detect a difference of 5% (SD 9%) in RVEF between arms; this difference is the MID for RVEF in PAH [35]. For HRQoL outcomes, we also have adequate power to detect clinically relevant changes in SF-36 of 5 units (SD 7 units) and emPHasis-10 of 6 units (SD 10) [36].

#### Data analysis plan

The intent-to-treat analysis will include all randomized subjects. Hypothesis testing will use two-sided α = 0.05 without correction for multiplicity. We will characterize subjects regarding baseline and follow-up 6MWD and other endpoints. We will summarize demographics and other predictors of clinical status. Continuous variables will be summarized by the mean, median, standard deviation, and range, as appropriate. The distribution of the outcome measures of interest will be assessed for normality. If the distribution is normal, we will use independent t-tests to compare by treatment assignment. If the distribution is not normal, we will transform the data to meet the assumption of normality. If transformation does not achieve normality, comparisons between groups will be made using the Wilcoxon rank-sum test. We will use contingency tables for discrete and dichotomous variables.

The primary analysis will compare the absolute change in 6MWD from baseline with adjustment for baseline 6MWD, age, and sex in linear regression models. We chose sex as a potential confounder of the relationship between 6WMD and treatment based upon prior work demonstrating there may be worse survival for men with SSc-PAH compared to women [37], but men may have improved 6MWD and HRQoL compared to women in response to PDE-5 inhibitors [38]. Given the small sample size of our study, we will be sensitive to overfitting our model with covariates but will use the univariate analyses to inform the inclusion of additional covariates in the final model. We will examine the clinical relevance of the change in 6MWD by comparing the proportion of patients who achieve the MID for the 6MWD for both PAH (MID = 33 m) and for SSc-PAH (MID = 24 m) by treatment assignment. Using separate multivariable logistic regression models adjusting for baseline walk distance, age, and sex, we will assess if the odds of achieving a clinically relevant improvement in 6MWD based upon the MID for PAH and for SSc-PAH differs by treatment assignment. We can then perform responder analyses to determine characteristics of SSc-MEP patients likely to achieve either MID for the primary outcome of 6MWD.

The secondary outcomes will be analyzed similarly using linear regression models adjusting for baseline values and for sex. Prior studies have demonstrated sex-specific differences in RV structure and function as assessed by CMR [39] and in PAC [29], though to our knowledge there are no studies examining differences in TAPSE between men and women. HRQoL measures may differ by sex as well.

We will also include longitudinal models of change from baseline over the time of the trial. Exploratory multivariate analyses will be performed incorporating all the available endpoint assessments (baseline, 4 months, and 12 months) in an ANCOVA model with active treatment/placebo status as the independent variable.

We will attempt to minimize missing data by encouraging full subject participation and follow-up even if the subject stops the study drug prematurely. If there are missing data, we will perform sensitivity analyses using the mean value obtained to replace this missing data. For subjects lost to follow-up, we will use all the information available until the end of follow-up. For dropouts, we will use the lowest value obtained to replace missing data in separate sensitivity analyses.

### Data monitoring

The Data Safety and Monitoring Board (DSMB) will be comprised of three members: two pulmonary hypertension specialists with extensive track records of participating in and leading randomized controlled trials in PH, as well as one biostatistician experienced in clinical trial analysis. DSMB reports will be generated on a quarterly basis including expected and actual enrollment numbers. Every six months, a DSMB report will be generated that includes enrollment rates, follow-up rates, compliance levels, adverse events, mortality, and mean data on the primary and secondary analysis. If there are any complaints about the research trial, we will summarize these complaints and report them to the DSMB in these reports. Since this is a Phase II trial that would be helpful in supporting future studies of the intervention even if no difference is found between sildenafil and placebo, we have not planned for formal interim analyses for futility.

### Ethics approval and consent to participate

The study is being conducted in compliance with the principles of Declaration of Helsinki and informed consent is obtained from all the participants. For each consent process, study personnel discuss the details of the study, the risks and benefits, and the subject’s rights and responsibilities if they choose to participate in the trial and their right to refuse to participate. It is made clear that their clinical care will not be affected by their decision. Subjects are permitted to provide verbal consent over the phone prior to being scheduled for a screening visit. A consent script is provided, and documentation of verbal consent is noted. When the subject arrives for the screening visit, written consent is obtained.

Johns Hopkins University and Louisiana State University Health Sciences Center are relying on a single IRB (sIRB) of record (JHU) and obtain approval and reliance agreements. IRB approval was required at both enrolling sites prior to enrollment of the first patient. Protocol changes must be approved by the sIRB prior to implementation. Unanticipated problems posing risks to subjects or others will be reported to the sIRB. Reportable events include any event that could represent an unexpected serious adverse event (SAE), any AE that could lead to a change in the informed consent, information that changes the risks and benefits of the trial, a change in FDA labeling for sildenafil, breach of confidentiality, protocol violations that might place one or more participants at increased risk or might affect the rights of the subjects.

### Data management, sharing, and dissemination

Confidentiality is maintained by assigning each participant with a unique study number; no Protected Health Information (PHI) is recorded on study case report forms (CRF) or transmitted between study sites. We keep any potential identifiers separate from the participant’s CRF in a secure environment only accessible to study staff granted access to PHI. Only study staff approved by the IRB have access to study records, data, and specimens. Representatives from the funding organization, the Department of Defense, are eligible to review study records.

Clinical site personnel key in all study data into the data capture system directly. Other than the two questionnaires, study data is entered directly from the electronic medical record, rather than by use of paper CRF’s. The electronic data capture system that we are using is REDCap (https://www.project-redcap.org/), which is a secure, validated web application used by both Johns Hopkins and Louisiana State University Health Sciences Center to build and manage databases. The paper forms (questionnaires) are kept in locked offices only accessible by approved study staff. To ensure data quality, all data is double-entered (enter/verify). A second method of quality control is embedded within REDCap, which provides data checks in real time as data are keyed, including data format checks (e.g., numbers or letters) and valid value checks (e.g., ranges for age). The site Principal Investigator (PI) performs continuous monitoring of data quality and completion of CRFs. Once the site personnel mark a form as complete, the data on that form is locked and all error corrections must be requested. Each time an error correction is done after the data form is locked, a rationale must be provided. Data will be exported only in de-identified custom exports and only by approval of the PI.

Results of this clinical trial will be disseminated to the research community through presentations at national/international conferences as well as through publications in peer-reviewed journals. Data collected during this clinical trial will be shared, both in aggregate through manuscripts and individual-level (de-identified) data upon request. The data will be encoded using standard methods and a coding key will be available to interested researchers. Additionally, biospecimens may be available for sharing if adequate samples remain after our analyses are complete. In this case, a Materials Transfer Agreement would be required according to institutional policy. Data will be distributed directly to interested researchers by request via an electronic file. Data may also be submitted to the NHLBI-funded Biologic Specimen and Data Repository Information Coordinating Center (BioLINCC), as the biomarker portion of our trial aligns with their mission.

## Discussion

To our knowledge, once completed the SEPVADIS trial will be the first RCT to exclusively enroll patients with mildly elevated mean pulmonary artery pressure (21-24mmHg). Since the recommended definition of PH changed in 2018, there has been great uncertainty about whether PAH-specific medications are beneficial in this new subgroup of patients, especially in a high-risk group such as SSc. The SEPVADIS trial is currently enrolling patients at both clinical sites and once completed will provide important data on the impact of sildenafil on exercise capacity, right heart function, and HRQoL in SSc patients with mildly elevated pulmonary artery pressure. We anticipate that these data will lay the foundation for a larger, multi-center trial of PAH-specific medications in SSc-MEP, which has the potential to slow progression of disease and positively impact patient outcomes.

## Data Availability

N/A.

## Abbreviations

6MWD: six-minute walk distance
6MWT: six-minute walk test
ACR: American College of Rheumatology
ANCOVA: analysis of covariance
BP: blood pressure
CI: Cardiac index
CMR: cardiac magnetic resonance imaging
CRF: case report form
CTA: chest CT angiography
DSMB: Data Safety and Monitoring Board
ERA: endothelin receptor antagonist
EULAR: European League Against Rheumatism
FC: functional class
FDA: Food and Drug Administration
HRCT: high resolution computed tomography
HRQoL: health-related quality of life
ILD: interstitial lung disease
JHU: Johns Hopkins University
LVEF: left ventricular ejection fraction
m: meter(s)
mmHg: millimeters of mercury
MEP: mildly elevated pulmonary pressure
MID: minimal important difference
mPAP: mean pulmonary artery pressure
NHLBI: National Heart, Lung, and Blood Institute
NT-proBNP: N-terminal B-type natriuretic peptide
PAC: pulmonary artery compliance
PAH: pulmonary artery hypertension
PAWP: pulmonary artery wedge pressure
PDE-5: phosphodiesterase-5
PH: pulmonary hypertension
PHI: Protected Health Information
PI: Principal Investigator
PVR: pulmonary vascular resistance
RCT: randomized controlled trial
RHC: right heart catheterization
RV: right ventricle
SAE: serious adverse events
SD: standard deviation
SEPVADIS: **S**ildenafil versus placebo for **e**arly **p**ulmonary **v**ascular **d**isease **i**n **s**cleroderma
SF-36: Short Form 36 questionnaire
sIRB: single institutional review board
SSc: systemic sclerosis
STE: speckle tracking echocardiography
TAPSE: tricuspid annular planar systolic excursion
VQ: ventilation-perfusion scan
WHO: World Health Organization
WSPH: World Symposium on Pulmonary Hypertension
